# Improving the Flame Retardant Efficiency of Layer by Layer Coatings Containing Deoxyribonucleic Acid by Post-Diffusion of Hydrotalcite Nanoparticles

**DOI:** 10.3390/ma10070709

**Published:** 2017-06-27

**Authors:** Federico Carosio, Jenny Alongi, Chiara Paravidino, Alberto Frache

**Affiliations:** 1 Dipartimento di Scienza Applicata e Tecnologia, Politecnico di Torino, Alessandria Campus, Viale Teresa Michel 5, 15121 Alessandria, Italy; federico.carosio@polito.it (F.C.); chiara.paravidino@studenti.polito.it (C.P.); alberto.frache@polito.it (A.F.); 2 Dipartimento di Chimica, Università degli Studi di Milano, Via Golgi 19, 20133 Milano, Italy

**Keywords:** layer by layer, cotton, flame retardancy, combustion, hydrotalcite, nanoparticles

## Abstract

This work deals with the use of hydrotalcite nanoparticle post-diffusion in layer by layer (LbL) coatings with the aim of improving their flame retardant action on cotton. The selected LbL components, which encompass polydiallyldimethylammonium chloride and deoxyribonucleic acid, aim at the deposition of an intumescent coating. Infrared spectra pointed out a super-linear growth of the investigated assembly, indicating the ability to deposit thick coatings while maintaining a relatively low deposition number. A post-diffusion process, performed by exposing the LbL-treated fabrics to two different concentrations of hydrotalcite water suspensions (0.1 or 1 wt %), was carried out to improve the fireproofing efficiency of these coatings. Coatings treated with the lowest concentration suspension partially swelled as a consequence of their structural rearrangements while the use of the highest concentration led to nanoparticle aggregates. Horizontal flame spread tests were used for assessing the achieved flame retardant properties. The post-diffusion performed at the lowest hydrotalcite concentration lowers the minimum number of Bi-Layers required for obtaining cotton self-extinguishment while samples treated with the highest concentration showed detrimental effects on the performances of treated fabrics. This behavior is ascribed to the effects of hydrotalcite particles on the intumescence of LbL coatings, as evidenced by the morphological analyses of post-combustion residues.

## 1. Introduction

In recent years, layer by layer (LbL) assembly has attracted the interest of the scientific community as a powerful tool for the surface modification of materials capable of constructing finely controlled nanocoatings. The fundamentals of this deposition technique can be dated back to the 1960s when Iler demonstrated that it was possible to build a coating on a glass surface by alternately exposing the surface to oppositely charged micro particles suspensions. Later, during the 1990s, this concept was reinvented and adapted to polyelectrolytes, leading to the modern definition of layer by layer assembly [1].

The technique is based on the alternate adsorption on a substrate of polyelectrolytes, nanoparticles, etc., on the basis of a specific interaction occurring in between the selected chemical species [2]. Up to now, the most widely employed interaction is the electrostatic attraction occurring between nanoparticles or polyelectrolytes in aqueous media; in addition, other interactions have been exploited such as donor/acceptor [3], hydrogen bonds [4] and covalent bonds [5].

Through the years, the layer by layer technique has been used for the construction of coatings; it is capable of conferring many different properties such as improved gas barrier, anti-reflection, super-hydrophobicity, antibacterial and flame retardancy [2,6,7].

**Figure 1:**
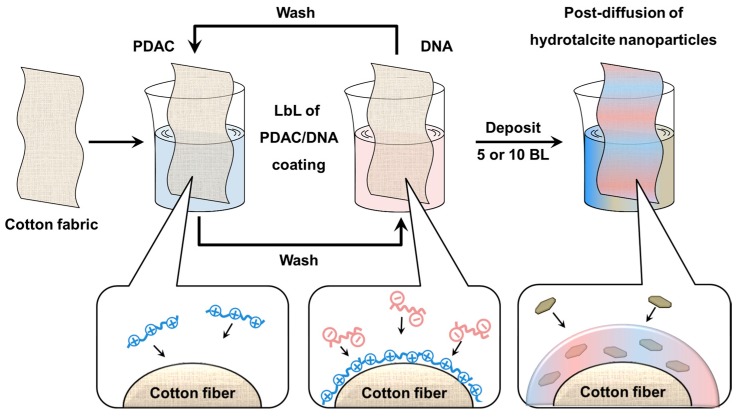
Schematic representation of the adopted layer by layer (LbL) assembly. Cotton fabrics are alternatively dipped in the positive polydiallyldimethylammonium chloride (PDAC) and negative deoxyribonucleic acid (DNA) solutions until 5BL or 10Bi-Layers (BL) are deposited. Then the coated fabrics are dipped into a hydrotalcite suspension for the post-diffusion process.

The properties achieved by LbL surface nanostructuring have been demonstrated to outperform those of other nanostructured materials. As an example, the fine control over the nanostructure and the preferential orientation of clay nanoplatelets in LbL coatings allowed for obtaining impressive gas barrier properties when LbL-coated polymers are compared with clay-containing polymer nanocomposites produced by melt blending [8,9]. Another example is represented by the flame retardancy field where the needs for novel, efficient and sustainable alternatives to some of the high performing and unfortunately toxic flame retardants have paved the way to the green and water-based LbL approach [10,11]. This latter approach has proven to be extremely promising and crucial in order to positively affect the burning behavior of a polymer. Indeed, by controlling the mass, oxygen and heat transfers at the interface between the gas and condensed phases, the surface can play a fundamental role in delaying ignition and/or slowing down combustion of a polymer [7]. Thus, the LbL has been successfully used for the design and construction of fire retardant coatings deposited on fabrics [12,13,14,15], open cell polyurethane foams [16,17,18], close cell polyester foams and thin films [19,20,21,22]. Thanks to the versatility of the LbL technique, the flame retardant action has been continuously modified and improved, starting from thermal-shield coatings completely made by inorganic nanoparticles moving to coatings with intumescent-like compositions, eventually combining these two flame retardant actions in hybrid coatings. In the latter approach, nanoparticles have been incorporated in intumescent-like coatings by introducing the adsorption of positively or negatively charged nanoparticles in the LbL sequence either as inorganic support during the first deposition steps or as inorganic reinforcement through the entire deposition process [23,24]. Another way to incorporate nanoparticles in a LbL coating is represented by the use of a post-diffusion treatment; here, nanoparticles can diffuse inside a previously deposited all-polyelectrolyte LbL coating when exposed to the nanoparticle suspension [25,26]. Although this strategy has proven to be an efficient way to load nano-objects inside a previously deposited LbL assembly, to the best of our knowledge, this approach has never been used for the deposition of flame retardant nanoparticle-reinforced LbL coatings. Thus, in the present paper, we are exploiting the LbL approach of an intumescent coating consisting of polydiallyldimethylammonium chloride (PDAC)/deoxyribonucleic acid (DNA) followed by the post-diffusion of inorganic hydrotalcite (HT) nanoparticles for the deposition of fireproofing coatings on cotton fabrics, as schematically depicted in Figure 1.

The coating composition has been selected in order to couple a strong polyelectrolyte, already employed in post-diffusion treatments [26], with an all-in-one intumescent component such as DNA, as recently demonstrated by our research group [27,28]. The use of HT as nanoparticles is linked to previous studies conducted by our research group that proved HT to be among the most promising flame retardant-oriented nanoparticles [29]. In the present study, two concentrations (namely 0.1 or 1 wt %) have been selected in order to evaluate the effect of nanoparticle concentration during the post-diffusion process. The coating growth has been followed by infrared spectroscopy (IR); the changes of cotton surface morphology after the LbL deposition and post-diffusion process have been evaluated by Scanning Electron Microscopy (SEM). The achieved flame retardant properties have been assessed by horizontal flame spread tests.

## 2. Results and Discussion

### 2.1. Coating Growth and Morphology on Cotton

The assembly growth of the PDAC/DNA pair has been assessed by infrared spectroscopy. First of all, the spectrum of each polyelectrolyte has been evaluated (Figure S1 in Supplementary Materials). Neat PDAC shows the most intense signal at 1477 cm^−1^ generally ascribed to both C–H deformation vibrations in CH_2_ and CH_3_ groups and O-H asymmetrical deformation of adsorbed water. In addition to the latter, the O–H symmetrical deformation signal is found at 1640 cm^−1^ [30,31]. The peak at 1136 cm^−1^ accounts for the C–N symmetrical stretching. The phosphate deoxyribose backbone of DNA shows the characteristic peaks located at 1232, 1063 and 967 cm^−1^ and ascribed to P=O and PO_2_^−^, C–O–C and P–O–C groups, respectively [32]. The C=C and C=N stretching vibrations of pyrimidine and purine bases are found in the 1500–1350 cm^−1^ region while the most intense signal at 1695 cm^−1^ is ascribed to the C=O vibration of guanine, cytosine and thymine. The two components have been LbL-assembled following the procedure described in Figure 1.

**Figure 2:**
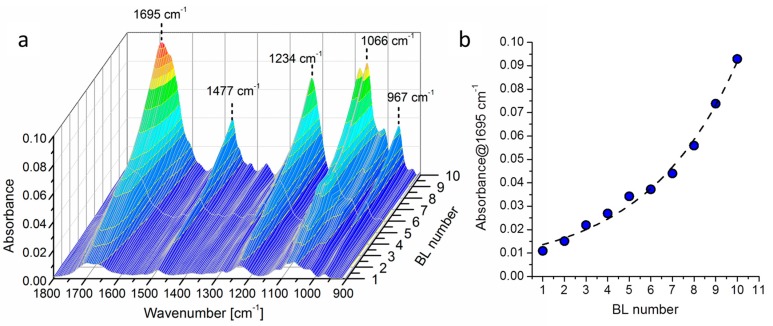
Coating growth followed by IR spectroscopy: (**a**) 3D projection of peaks present in the 1800–900 cm^−1^ region and (**b**) intensity of signal at 1695 cm^−1^ as a function of BL number.

Figure 2 reports the elaboration of IR signals of the PDAC/DNA system growth on a model Si wafer substrate and the intensity plot of the 1695 cm^−1^ peak as a function of BL number.

When the two components are LbL-assembled on Si wafer, the main signals ascribed to DNA (967, 1066, 1234 and 1695 cm^−1^) and PDAC (1477 cm^−1^) are clearly visible and grow proportionally to the number of deposited layers, as reported in Figure 2a. It is then possible to assess the growth regime of the assembled coating by evaluating the change in intensity of one characteristic peak; to this aim, the intensity of the peak at 1695 cm^−1^ as a function of BL number has been plotted in Figure 2b. The plot clearly points out a super-linear growth regime for PDAC/DNA assembly. This is in accordance with the previous literature on LbL dealing with chitosan/DNA coatings [33]. Such super-linear growth is very useful as it allows for the deposition of thick coatings even at low BL numbers, thus improving the efficiency of the post-diffusion process adopted in this work.

Later on, the coating was deposited on cotton fabrics in order to improve its flame retardancy. Cotton represents an ideal substrate for the LbL deposition due to its high hydrophilicity and intrinsically roughness and uneven morphology of its fibers, as demonstrated by the SEM micrograph reported in Figure S2.

The 5BL and 10BL coatings have been deposited on cotton; after the deposition, the so-treated fabrics were dipped into a hydrotalcite (HT) suspension at two different concentrations (0.1 and 1 wt %). Table 1 lists sample codes and weight gain with the description of treatment conditions.

**Figure 3:**
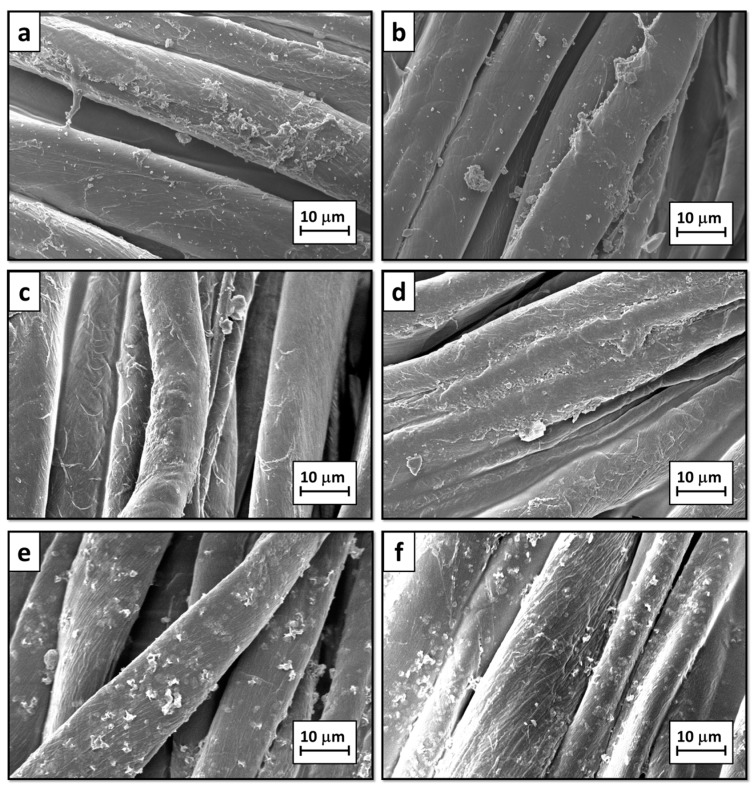
SEM micrographs of treated cotton fabrics: (**a**) 5BL; (**b**) 10BL; (**c**) 5BL + 0.1%_HT; (**d**) 10BL + 0.1%_HT; (**e**) 5BL + 1%_HT and (**f**) 10BL + 1%_HT.

The changes of surface morphology after the LbL deposition (5BL and 10BL samples) and post-diffusion process (5BL + 0.1%_HT, 10BL + 0.1%_HT, 5BL + 1%_HT, and 10BL + 1%_HT samples) have been observed by SEM; micrographs of so-treated fabrics are reported in Figure 3.

The deposition of 5BL and 10BL gradually changes the cotton surface morphology. The formation of a smooth coating can be easily observed in Figure 3a,b; the best surface coverage is obtained when 10BL are present while when 5BL are present some untreated areas can still be spotted. Both 5BL and 10BL samples show the presence of small debris likely ascribed to the coating. After the post-diffusion of HT from a 0.1 wt % suspension, the samples show no significant changes in surface morphology (Figure 3c,d); however, the coatings appear swollen and less smooth than before, indicating a possible structural change after HT-post-diffusion. This swelling is particularly clear when comparing the micrographs of the 10BL coatings before and after nanoparticle diffusion (compare Figure 3b with Figure 3d). By increasing the concentration of the HT suspension to 1 wt %, it is possible to significantly increase the amount of post-diffused nanoparticles. This leads not only to the coating swelling, as observed before, but also to the formation of HT aggregates that are found on top of the LbL coating (Figure 3e,f). Such aggregates are homogeneously distributed on the surface of the coated fabric, as demonstrated by low magnification micrographs that further point out the morphological differences between LbL-coated samples post-treated with 0.1 or 1 wt % HT suspensions (Figure S3).

### 2.2. Flammability

**Figure 4:**
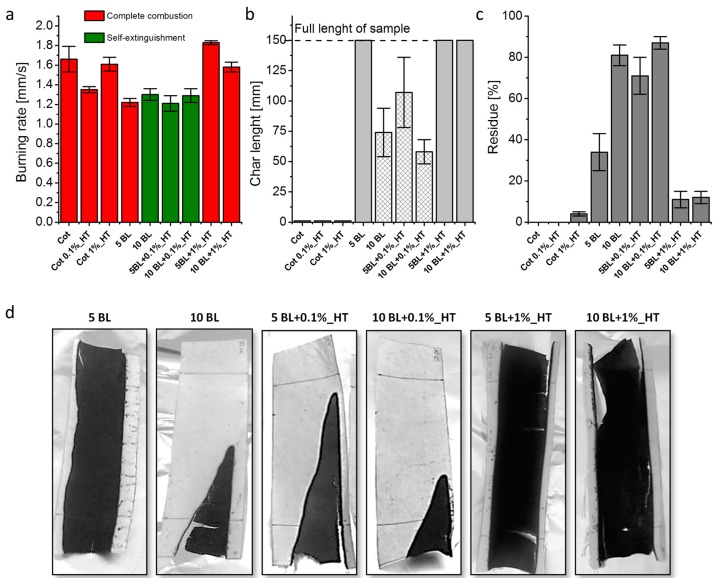
Combustion data from horizontal flame spread tests: (**a**) burning rates; (**b**) char length; (**c**) residue and (**d**) pictures of the residues at the end of the test.

The horizontal flame spread test has been employed in order to assess the achieved flame retardancy properties. This test evaluates the reaction of untreated and treated fabrics to a direct methane flame application, eventually providing information on the propensity of the tested sample to initiate and/or spread a fire. Figure 4 reports the parameters collected during the tests and the images of the post-combustion residues. In order to properly evaluate the effect of HT post-diffusion treatment on LbL treated fabrics, other samples have been prepared by single step dipping into a 0.1 and 1 wt % HT suspension, as well (namely, Cot 0.1%_HT or Cot 1%_HT samples in Table 1).

Upon methane flame application, untreated cotton immediately ignites and vigorously burns. The flame quickly spreads to the opposite edge of the sample without leaving any residue. A similar behavior has been observed for cotton fabrics treated only with HT suspensions. The presence of HT alone is not enough to consistently change the burning behavior of cotton; a reduction in burning rate is observed only for 0.1 wt % samples (Figure 4a). On the other hand, while untreated cotton and cotton treated with 0.1 wt % HT left no residue at the end of the test, samples treated with 1 wt % yielded a slightly increased residue (4% as reported in Figure 4c) in the form of light ashes (see Figure S4).

Conversely to HT simple adsorption, the presence of the PDAC/DNA LbL coating dramatically changes the burning behavior of cotton. The 5BL allow for the reduction of the burning rate and the formation of a coherent and compact residue (34%) accounting for the entire length of the sample, as reported in Figure 4b and depicted in Figure 4d. On the other hand, 10BL exhibit self-extinguishing behavior. After ignition, the flame spreads but at the same time is gradually reduced in size and it is forced to a progressively smaller portion of the fabric where it eventually extinguishes (Figure 4d). For this reason, the final residue is as high as 81% (Figure 4c).

The post-diffusion of HT particles from a 0.1 wt % suspension is capable of improving the performances of LbL coating. Indeed, as reported in Figure 4, the self-extinguishing behavior is now observed after 5BL while at 10BL the flame extinguishes in a reduced space (compare char length values in Figure 4b and residue pictures in Figure 4d). Conversely, by increasing the concentration of the HT suspension to 1 wt %, a detrimental effect is observed. Indeed, these latter samples burned completely yielding burning rates similar to untreated cotton and decreased final residues (11-12%).

### 2.3. Cone Calorimetry

Cone calorimetry has been employed in order to provide information complementary to the flammability test. This technique allows for evaluating the behavior of the fabrics when exposed to the heat flux normally found in developing fires (namely, 35 kW/m^2^). During the test, the exposure to the heat flux triggers the thermal degradation of the samples in air that starts releasing combustible volatiles. At sufficient concentration, these gaseous species are ignited by a spark igniter, leading to the flaming combustion of the sample. The heat released is then calculated by measuring the oxygen consumed during combustion. Table 2 reports the main combustion parameters normally assessed by cone calorimetry.

As reported in Table 2, neat cotton ignites after 29 s with an average peak of Heat Release Rate (pkHRR) of 109 kW/m^2^ and a Total Heat Release (THR) of 1.9 MJ/m^2^. The only benefit provided by simple impregnation with hydrotalcite is a slight increase in the time to ignition (TTI) proportional to the employed clay concentration, while the other parameters remain almost unchanged or within the experimental errors. On the other hand, the combustion kinetics is affected by the deposition of the PDAC/DNA LbL coating, as demonstrated by the 20% and 21% reduction in pkHRR and THR values, respectively achieved by both 5BL and 10BL samples. The TTI is reduced similarly to previously reported LbL coatings containing DNA [33]; indeed, the phosphate groups of DNA lead to an anticipation of ignition by promoting the dehydration and char formation reactions of cellulose. This also allows thermally stable structures to be built up on the surface of the fibers, capable of slowing down mass and heat transfer, as demonstrated by the reduction of pkHRR and THR. The post-diffusion of HT nanoparticles improves this effect by further reducing the pkHRR and THR values by 33% and 27%, respectively (10 BL + 0.1%_HT). Again, as observed during flammability tests, the post-diffusion of nanoparticles at the lowest concentration allows for the best performances. Indeed, as reported in Table 2, the use of 1 wt % HT suspension provides similar benefits in terms of pkHRR reduction but does not affect THR values.

### 2.4. Analysis of Post-Combustion Char Residue

**Figure 5:**
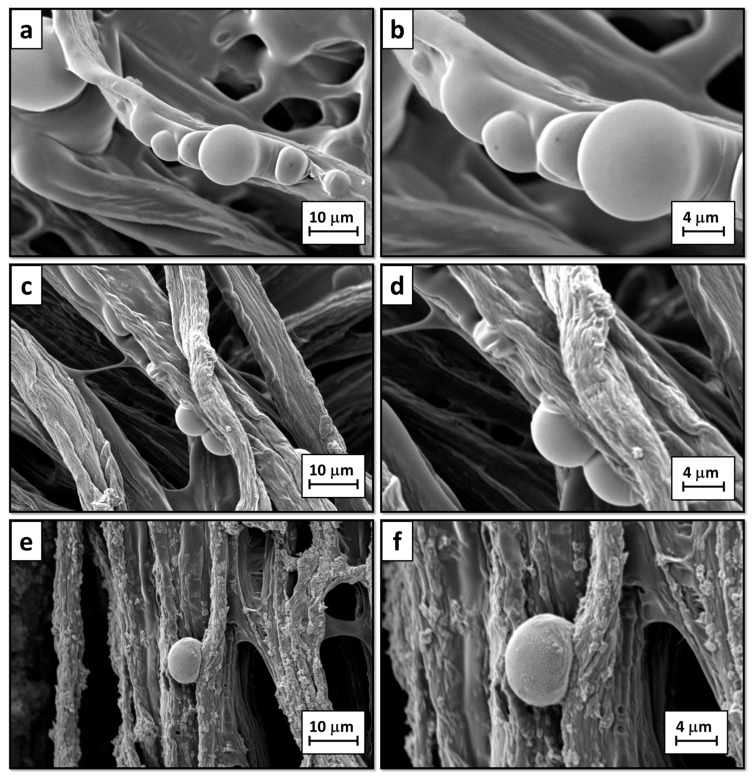
SEM micrographs of post-combustion residues: 10BL (**a**,**b**); 10BL + 0.1%_HT (**c**,**d**) and 10BL + 1% HT (**e**,**f**).

In order to further investigate the effects of the LbL coating and the post-diffusion process, the flammability residues of 10BL samples with and without post-diffusion have been collected and imaged by SEM. Figure 5 reports the acquired micrographs at different magnifications.

As reported in Figure 5a,b, 10BL residue shows the formation of intumescent bubbles that act with the double effect of slowing down the release of combustible volatiles and reducing the heat transmission towards the fabric. Similar morphologies have already been reported for chitosan/DNA coatings and ascribed to the intrinsic intumescent nature of DNA [34]. Indeed, the presence of nitrogen-containing bases has been found to be responsible for the release of ammonia as a blowing agent; in the particular case of PDAC/DNA assemblies, another source of gas is represented by the water adsorbed within the coating due to the presence of PDAC [35]. The 10BL treated with the post-diffusion from 0.1 wt % HT show similar morphologies, indicating that the presence of HT particles did not affect the intumescence mechanism of the deposited LbL coating (Figure 5c,d). Conversely, when the concentration of HT is increased up to 1 wt %, the number and size of intumescent structures is considerably reduced. Furthermore, the morphology of such structures is different from what was observed before as HT aggregates are on top of the few bubbles detected and the surface appears wrinkled (Figure 5f). The fibers appear more damaged and show reduced size with respect to the ones imaged in the other two residues (compare Figure 5b,d,f). This is also confirmed by low magnification micrographs that clearly show how samples treated with 1 wt % HT only partially maintained the original texture of the fabric (see Figure S5).

On the basis of flammability data and SEM observations of post-combustion residues, it is possible to depict the effect of the post-diffusion treatment on the flame retardancy performances of PDAC/DNA coatings. When the HT suspension is used at 0.1 wt %, the presence of the particles does not affect the intumescent behavior of the coating but can contribute to improving the overall flame retardant effect by mainly acting as a reinforcing agent of the expanded protective structures and by releasing noncombustible gases that dilute the volatile species released by cotton [36]. A catalytic effect on the intumescence process can also be considered, as already reported in the literature [37]. On the other hand, when HT is employed at 1 wt %, the formation of aggregates on top of the LbL coating has a detrimental effect as its intumescence behavior is significantly reduced. As a consequence, the coating cannot expand, as it normally would, thus it fails in building up an efficient and steady fire protection during combustion.

The flame retardant mechanism of such coatings can be summarized as follows. Upon exposure to a flame or a heat flux, the DNA embedded within the coating starts its degradation pathways where the phosphate groups enhance the dehydration of the deoxyribose backbone, resulting in the formation of thermally stable charred structures that are simultaneously expanded by the ammonia released by the nitrogen containing bases. The water embedded within the coating and the degradation products released from PDAC and cotton can also contribute to the expansion. These thermally stable structures act as a barrier, reducing the amount of cotton degradation products that feed the flame and, at the same time, the heat transmission from the flame to the bulk, thus resulting in a flame retardant effect. The post-diffusion of HT nanoparticles can enhance this barrier effect by providing an inorganic reinforcement to the expanded structure, releasing non-combustible gases and catalyzing the intumescence reaction. This joint effect allows for the best properties from both the flammability and cone calorimetry point of view.

## 3. Materials and Methods

### 3.1. Materials

Cotton with 200 g/m^2^ density area was purchased from Fratelli Ballesio S.r.l. (Torino, Italy). Prior to deposition, the fabrics were washed with a three-step process: (1) water and Marseille soap; (2) ethanol and (3) diethyl ether. At the end of the process, fabrics were dried in an oven at 70 °C for 1 h.

Branched poly(ethylene imine) (BPEI, M_w_ ~ 25,000 by Laser Scattering, M_n_ ~ 10,000 by Gel Permeation Chromatography, as reported in the material datasheet), poly(diallyldimethylammonium chloride) (PDAC, average M_w_ = 400,000–500,000, 20 wt %) and deoxyribonucleic acid sodium salt powder (DNA, from herring sperm) were purchased from Sigma Aldrich (Milwaukee, WI, USA). DNA was stored at 4 °C before its application to the fabrics. Commercially available ZnAl hydrotalcite (Zn_2_Al(OH)_6_(NO_3_)∙1.5H_2_O, HT) nanoplatelets, intercalated by nitrate ions were purchased from Prolabin & Tefarm (Università di Perugia, Perugia, Italy). Figure S6 reports a high magnification FESEM (Field Emission Scanning Electron Microscopy) micrograph of employed nanoplatelets.

All reagents were used as received for preparing water solutions/suspensions, using 18.2 MΩ deionized water supplied by a Q20 Millipore system (Milano, Italy). BPEI was employed at 0.1 wt %, PDAC and DNA at 1 wt %, and HT at 0.1 or 1 wt %. All solutions/suspensions were prepared at room temperature and left under magnetic stirring overnight.

### 3.2. Layer by Layer Deposition and Post-Diffusion Process

Prior to LbL deposition, Si wafers were dipped in a 0.1 wt % BPEI solution (5 min) in order to prime the surface. Then, the Si wafer or cotton fabrics were alternately immersed into the positively (PDAC) and the negatively (DNA) charged solutions; after each adsorption step, the excess solution was removed by washing and compressed air (Si wafers) or washing and vigorous squeezing (fabrics). The immersion period for the first couple of layers was set at 5 min; the subsequent layers were obtained after 1 min dipping. The process was repeated until 10BL (Bi-Layers) were built on Si wafer and 5BL or 10BL were deposited on cotton.

The post-diffusion of HT was performed by dipping the dried (5BL or 10BL) LbL-treated fabrics into the HT suspension for 30 min, following the experimental conditions already optimized elsewhere [35]. After this time, fabrics were squeezed and dried in a ventilated oven at 80 °C.

This process was also employed for the preparation of cotton fabrics treated with the simple impregnation of HT and tested as a comparison during flammability tests. Samples are coded as reported in Table 1 together with the weight gain and some details about the treatment.

### 3.3. Characterization of LbL Coatings and Treated Fabrics

Fourier-transformed infrared spectroscopy. The growth of the LbL assembly on the Si wafer was followed using a Frontier FT-IR/FIR spectrophotometer (16 scans and 4 cm^−1^ resolution, Perkin Elmer, Waltham, MA, USA). IR spectra are acquired after the deposition of each BL.

Scanning Electron Microscopy. The surface morphology of untreated, LbL-treated fabrics and post-combustion residues was studied using a LEO-1450VP Scanning Electron Microscope (imaging beam voltage: 5 kV, Bergen, NJ, USA). SEM imaging was performed on gold-metallized small samples (10 × 10 mm^2^) cut from the fabric or the post-combustion residue and fixed to conductive adhesive tapes. HT nanoparticles have been imaged using ZEISS, FEG model MERLIN.

Flammability. The flammability of untreated and treated fabrics was evaluated in horizontal configuration. The sample (150 × 50 mm^2^) is positioned in a metallic frame tilted by 45° along its longer axis, then ignited by a 20 mm methane flame (flame application time: 3 s). The test was repeated at least 3 times for each formulation in order to ensure reproducibility; during the test, parameters such as burning rate, char length and final residue were registered.

Cone calorimetry: an oxygen consumption cone calorimeter (Fire Testing Technology, FTT) was employed to investigate the combustion behavior of square samples (100 × 100 mm^2^) under 35 kW/m^2^ irradiative heat flux. Time to ignition (TTI), heat release rate and corresponding peak (pkHRR), total heat release (THR) and final residue were evaluated. The test was repeated 3 times for each formulation in order to ensure reproducibility; the experimental error was assessed as standard deviation (σ).

## 4. Conclusions

The present work presents, for the first time, the use of hydrotalcite nanoparticle post-diffusion in LbL assembled coatings in order to improve the flame retardant action. An all-polyelectrolyte LbL system encompassing polydiallyldimethylammonium chloride and deoxyribonucleic acid has been selected. IR spectroscopy pointed out the super-linear growth of selected polyelectrolytes that are thus capable of assembling thick coatings, even at relatively low BL number. The 5BL or 10BL of this system were then deposited on cotton and imaged by SEM, showing the formation of a homogeneous coating. The so-treated fabrics were exposed to hydrotalcite suspensions at two different concentrations (i.e., 0.1 or 1 wt %) in order to allow the post-diffusion of nanoparticles inside the LbL coating. SEM investigations demonstrated that coatings treated with the lowest concentration suspensions partially changed their morphology by swelling as a consequence of structural rearrangements due to the presence of rigid lamellar nanoparticles. On the other hand, the use of the highest concentration led to the formation of nanoparticle aggregates embedded within or found on top of the deposited coatings. The post-treatment with nanoparticle suspension at the lowest concentration improved the fireproofing action of the coating, allowing for a reduction of the deposition steps, capable of guarantying cotton self-extinguishment during horizontal flammability tests. Indeed, such post-treatment was found capable of lowering the minimum number of BL required for reaching cotton self-extinguishment (i.e., from 10BL to 5BL) while still improving the performances of the coatings deposited at a high BL number. Conversely, samples treated with the highest concentration suspensions showed a rather detrimental effect on the flame retardant performances of the LbL coating. This has been explained by an impeded intumescent behavior due to the presence of hydrotalcite aggregates on the top of LbL coatings, as assessed by SEM observations of the post-combustion residues. Similar results have been achieved during cone calorimetry tests where the deposition of 10BL followed by the post-diffusion from 0.1 wt % HT suspension yielded the best performances in terms of pkHRR and THR reductions. Compared to other LbL coatings containing chitosan and DNA, the proposed post-diffusion treatment allows for better flame retardant properties as self-extinguishment can be achieved with fewer layers deposited (i.e., 10 layers as reported in this paper *VS*. 20 layers of the previously published chitosan/DNA system) [33]. Other LbL assemblies, not containing DNA, have been shown to reach similar performances with only four or six deposition steps thanks to the use of polyphosphates in combination with chitosan or starch [12,14]. These latter systems represent potential candidates for use in combination with nanoparticle post-diffusion in order to further improve their flame retardant efficiency.

In conclusion, the described post-diffusion approach makes it possible for the development of new flame retardant strategies that are capable of improving the efficiency and performances of LbL coatings. The concepts described in this paper represent a step towards highly efficient and green flame retardant solutions that could possibly be exploited and extended to several substrates.

## Figures and Tables

**Table 1:** Codes, weight gain and description of the samples under study.

Code	Weight Gain (%)	Description
Cot	-	Untreated cotton
Cot 0.1%_HT	3	Cotton treated by simple impregnation of HT at 0.1 or 1 wt % suspension
Cot 1%_HT	7	
5BL	6	Cotton treated by 5 or 10BL of PDAC/DNA
10BL	13	
5BL + 0.1%_HT	6	Cotton treated by 5 or 10BL of PDAC/DANN followed by post-diffusion of HAT using 0.1 or 1 wt % suspensions
10BL + 0.1%_HT	14	
5BL + 1%_HT	8	
10BL + 1%_HT	15	

**Table 2:** Combustion data of untreated and LbL-treated cotton fabrics by cone calorimetry.

Sample	TTI ± σ (s)	pkHRR ± σ (kW/m^2^)	THR ± σ (MJ/m^2^)	Residue (%)
Cot	29 ± 6	109 ± 5	1.9 ± 0.1	0
Cot 0.1%_HT	32 ± 5	109 ± 4	1.9 ± 0.1	1
Cot 1%_HT	37 ± 3	95 ± 3	2.1 ± 0.2	2
5BL	15 ± 2	86 ± 2	1.5 ± 0.1	5
10BL	16 ± 1	87 ± 5	1.5 ± 0.1	6
5BL + 0.1%_HT	14 ± 2	82 ± 6	1.6 ± 0.2	8
10BL + 0.1%_HT	15 ± 4	73 ± 16	1.4 ± 0.2	10
5BL + 1%_HT	20 ± 2	71 ± 3	1.9 ± 0.2	7
10BL + 1%_HT	10 ± 2	81 ± 1	2.2 ± 0.2	4

## Supplementary Materials

The following are available online at www.mdpi.com/1996-1944/10/7/709/s1, Figure S1: FT-IR spectra of neat PDAC and DNA, Figure S2: SEM micrograph of untreated cotton, Figure S3: Low magnification SEM micrographs of (a) 5BL + 0.1%_HT, (b) 10BL + 0.1%_HT, (c) 5BL + 1%_HT and (d) 10BL + 1%_HT, Figure S4: Post-combustion residues of neat cotton and cotton treated with simple adsorption of HT nanoparticles, Figure S5: Low magnification SEM micrographs of post-combustion residues: (a) 10BL, (b) 10BL + 0.1%_HT, and (c) 10BL + 1%_HT, Figure S6: FESEM micrograph of employed HT.
