# Supplementary material for: Improving the Flame Retardant Efficiency of Layer by Layer Coatings Containing Deoxyribonucleic Acid by Post-Diffusion of Hydrotalcite Nanoparticles
Source: Materials (Basel). 2017 Jun 27;10(7):709. doi: 10.3390/ma10070709 (PMC5551752; doi:10.3390/ma10070709)
Supplement: Supplementary file 1 [file materials-10-00709-s001.pdf]

Supplementary Materials: Improving the Flame Retardant Efficiency of Layer by Layer Coatings Containing Deoxyribonucleic Acid by Post-Diffusion of Hydrotalcite Sanoparticles

Federico Carosio [1], Jenny Alongi [2,*], Chiara Paravidino [1] and Alberto Frache [1]

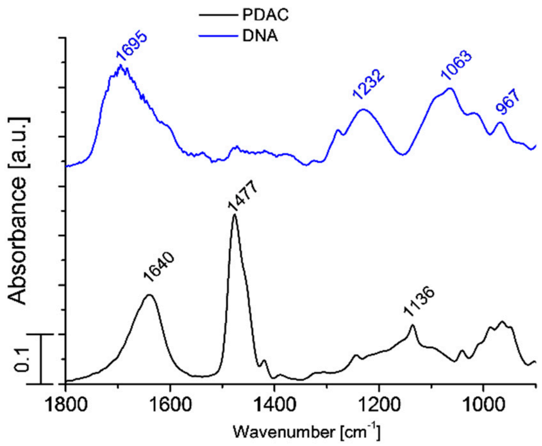

Figure S1. IR spectra of neat PDAC and DNA.

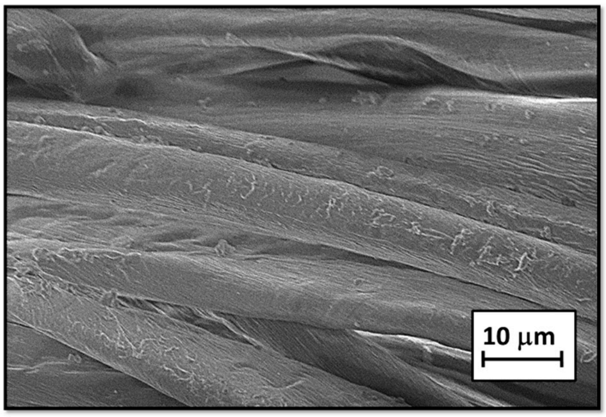

Figure S2. SEM micrograph of untreated cotton.

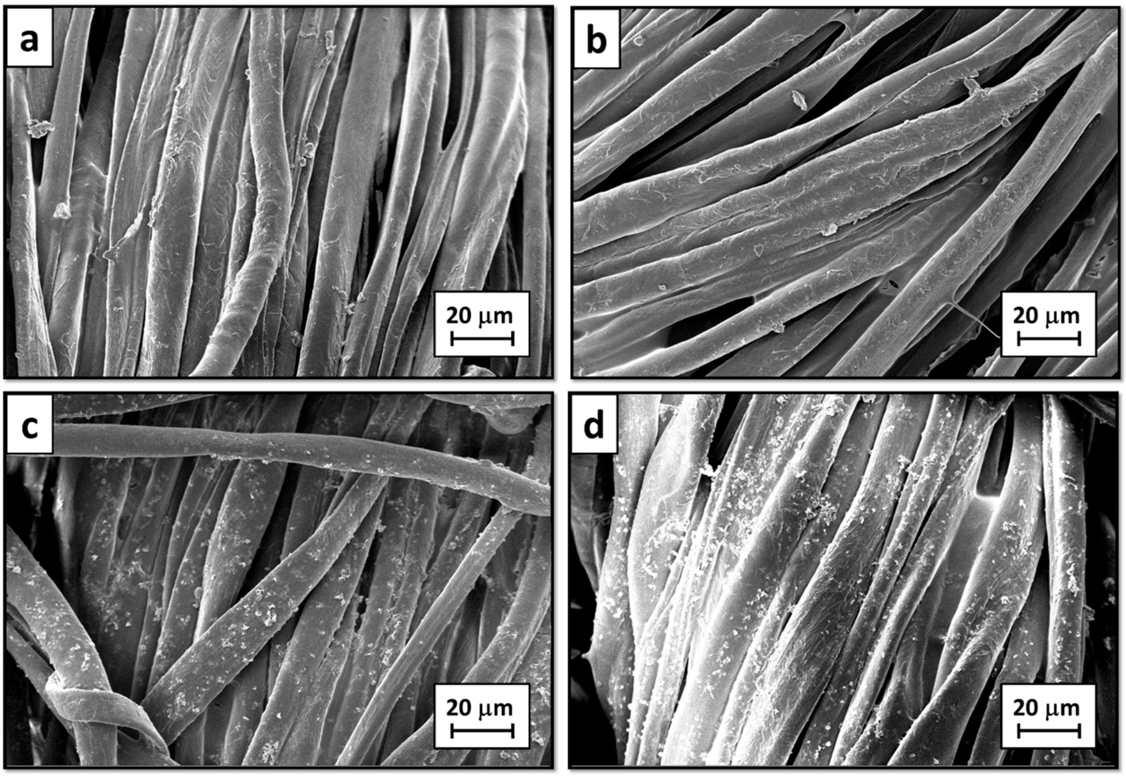

Figure S3. Low magnification SEM micrographs of (**a**) 5BL+0.1%_HT; (**b**) 10BL+0.1%_HT; (**c**) 5BL+1%_HT and (**d**) 10BL+1%_HT.

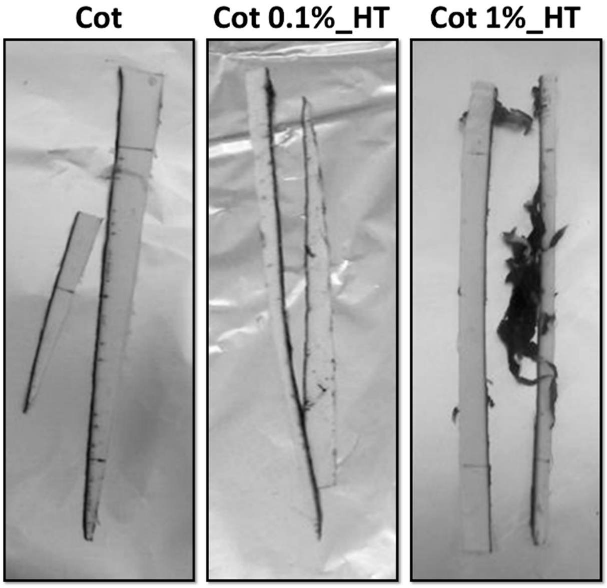

Figure S4. Post-combustion residues of neat cotton and cotton treated with simple adsorption of HT nanoparticles.

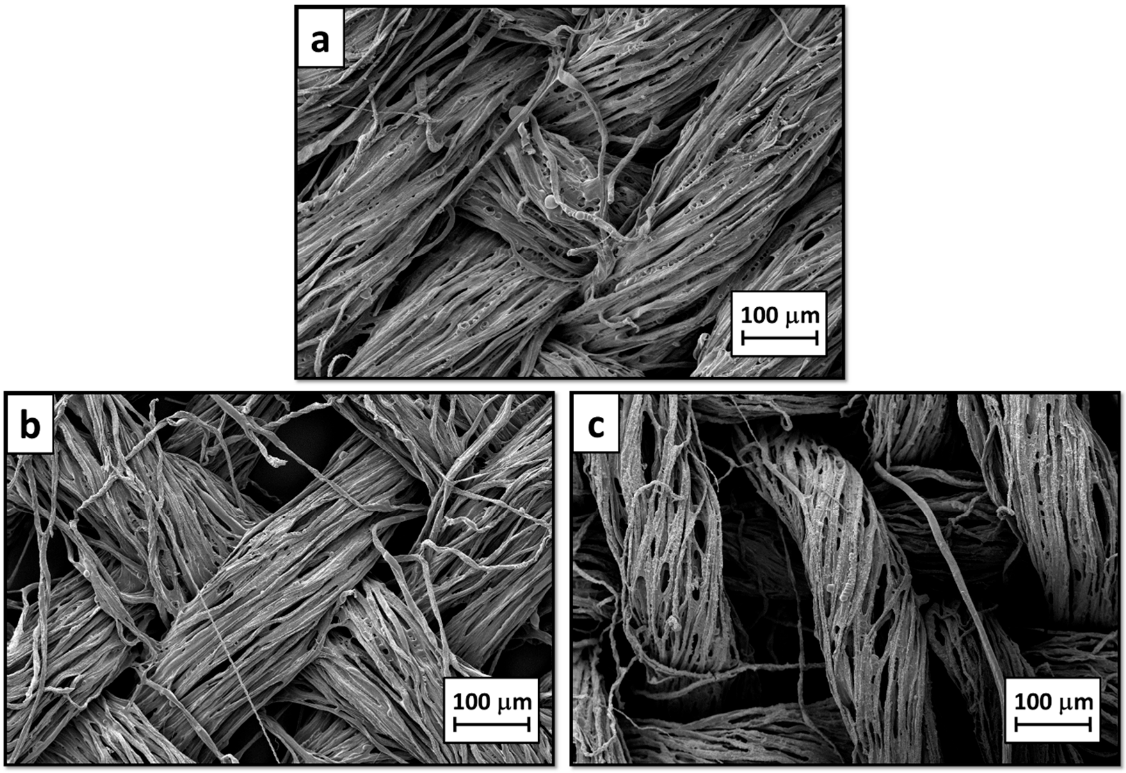

Figure S5. Low magnification SEM micrographs of post-combustion residues: (**a**) 10BL; (**b**) 10BL+0.1%_HT and (**c**) 10BL+1%_HT.
